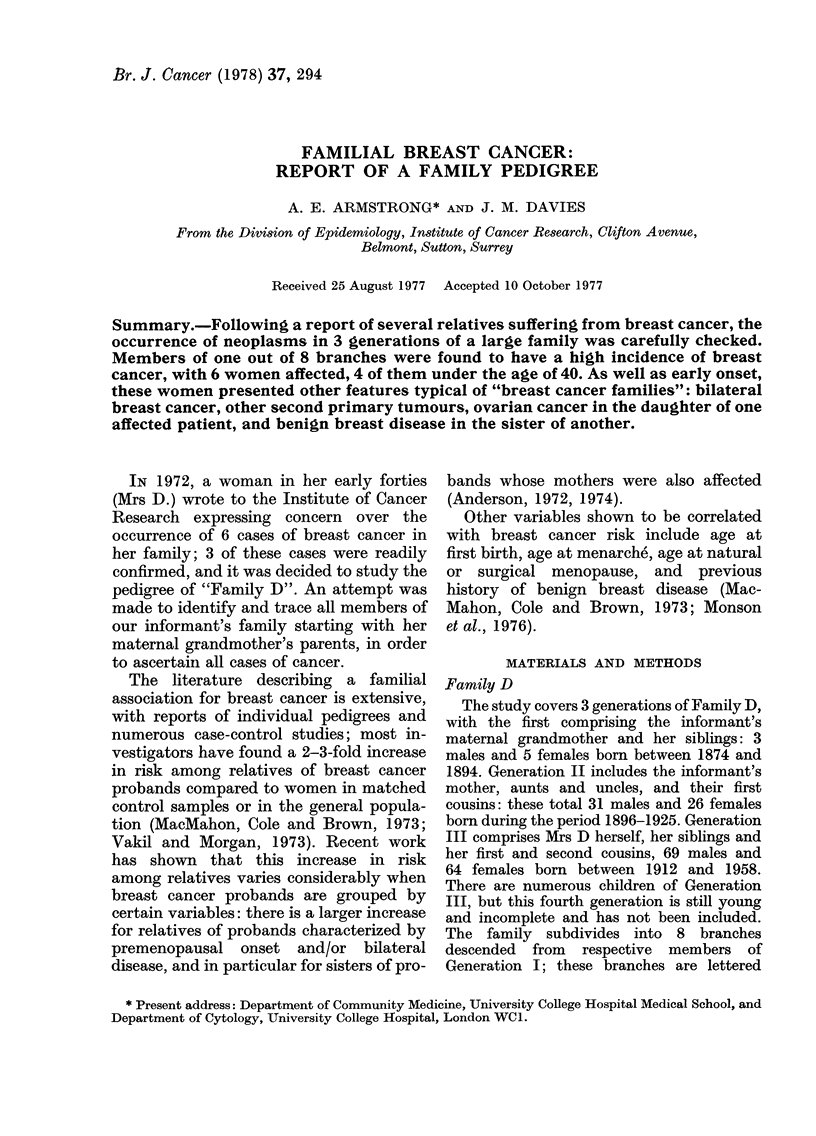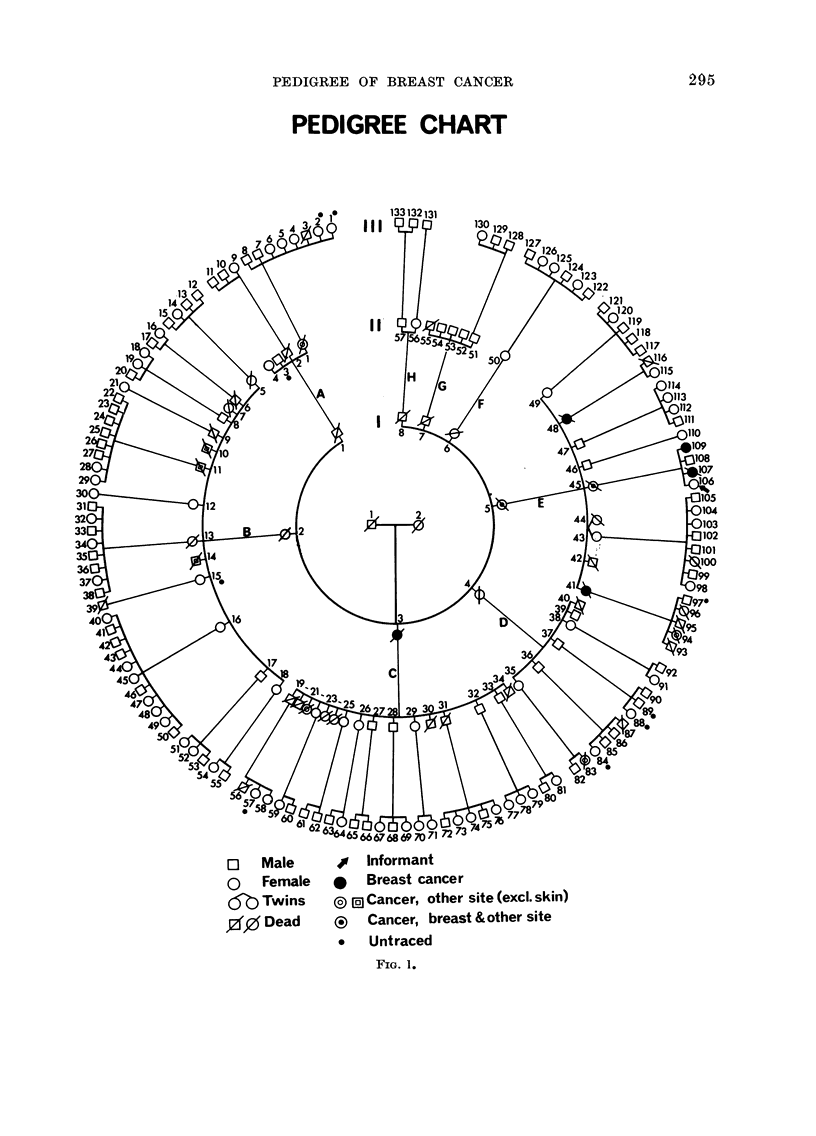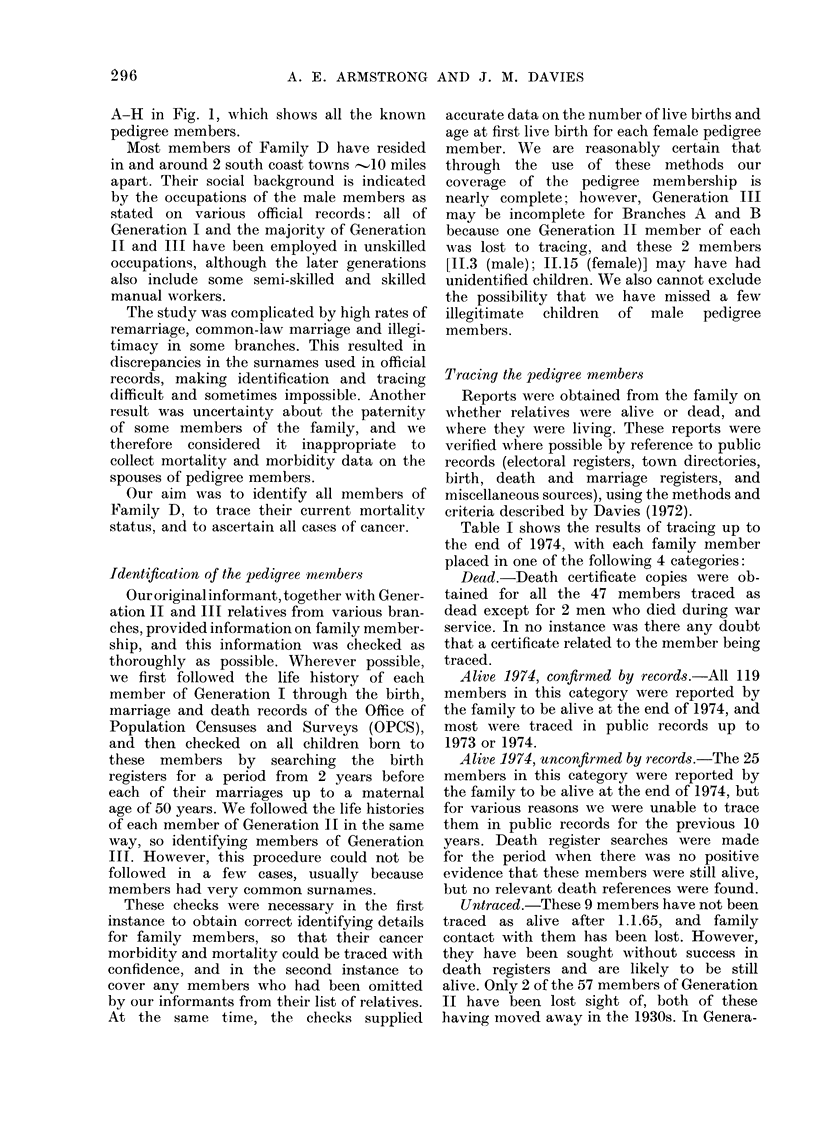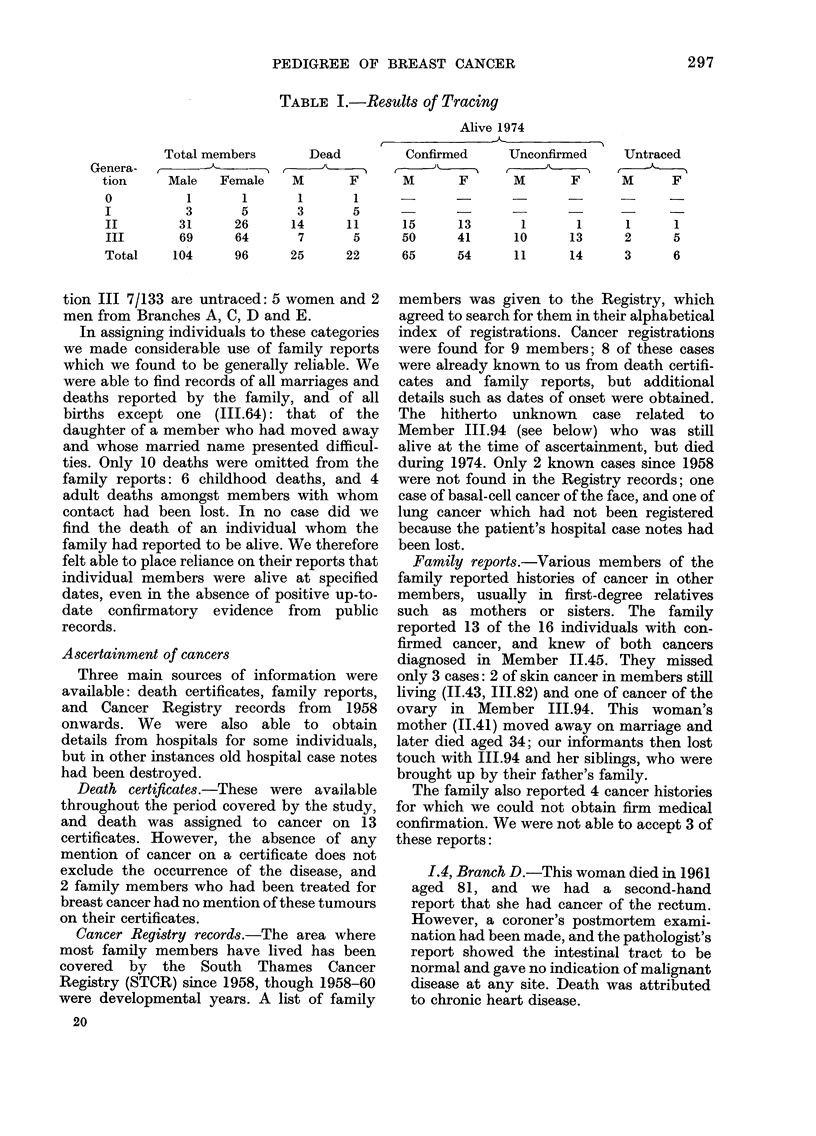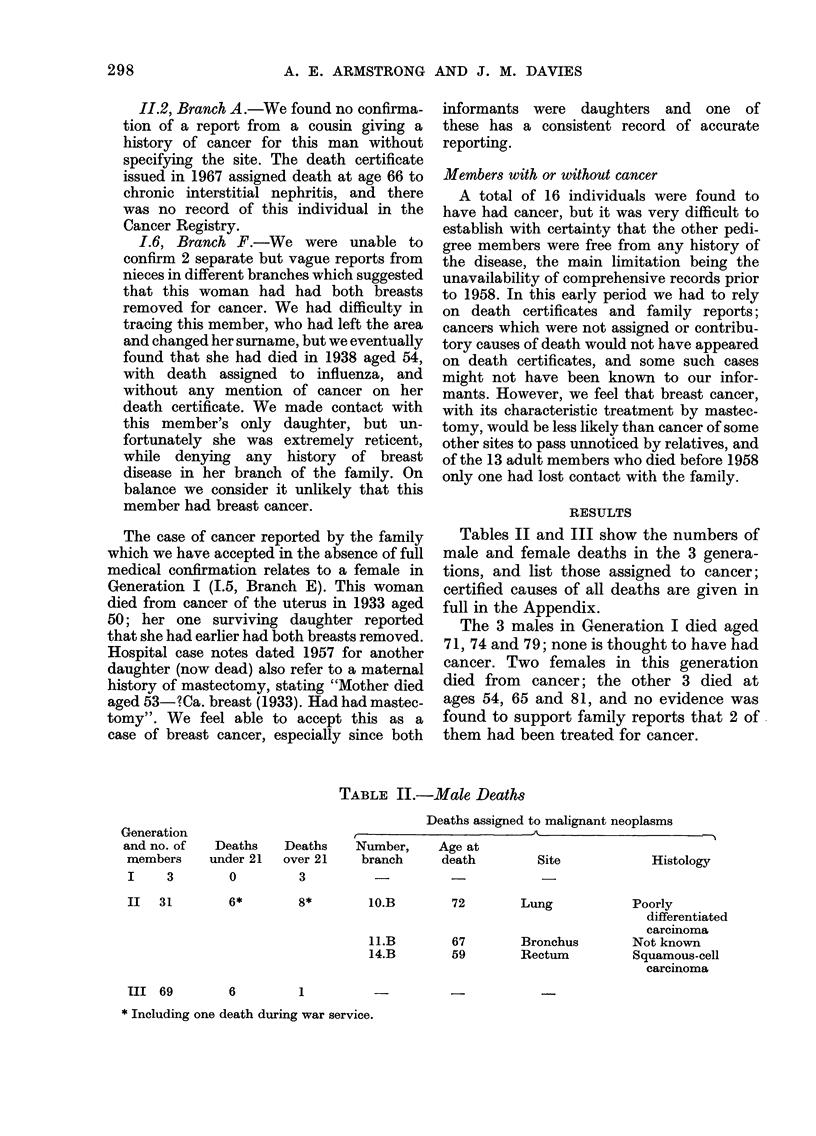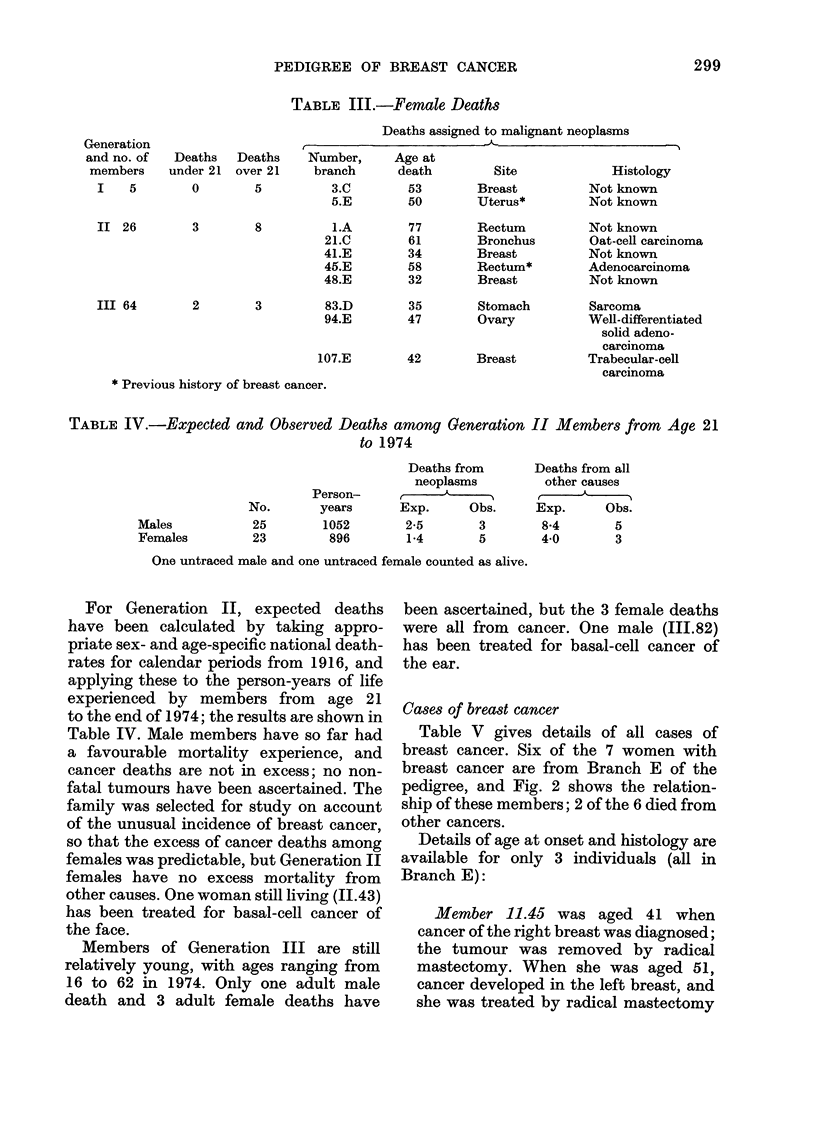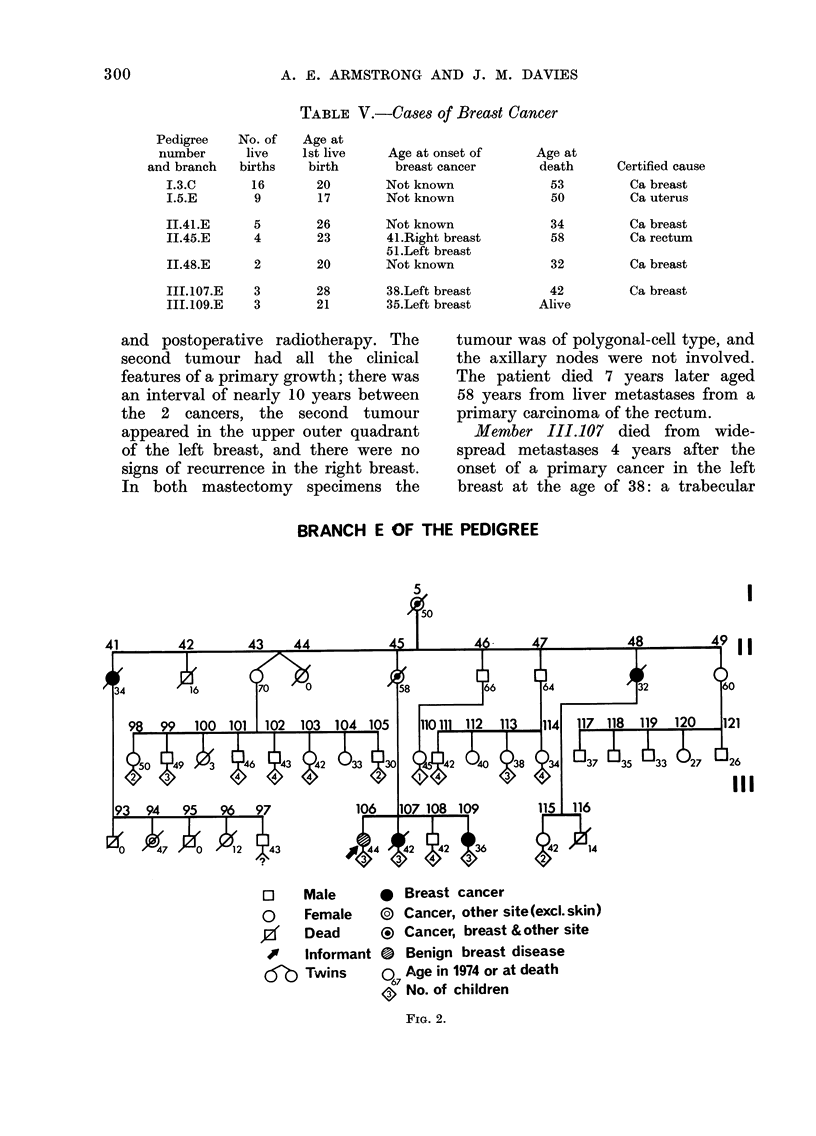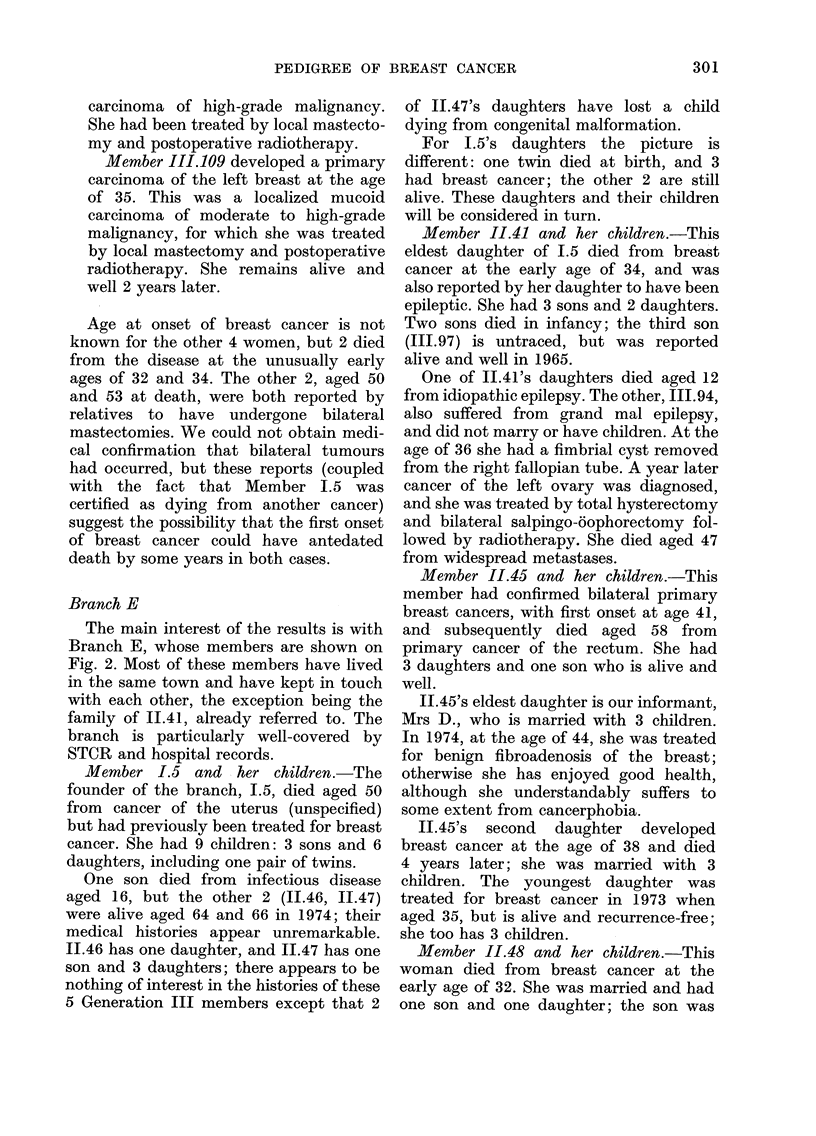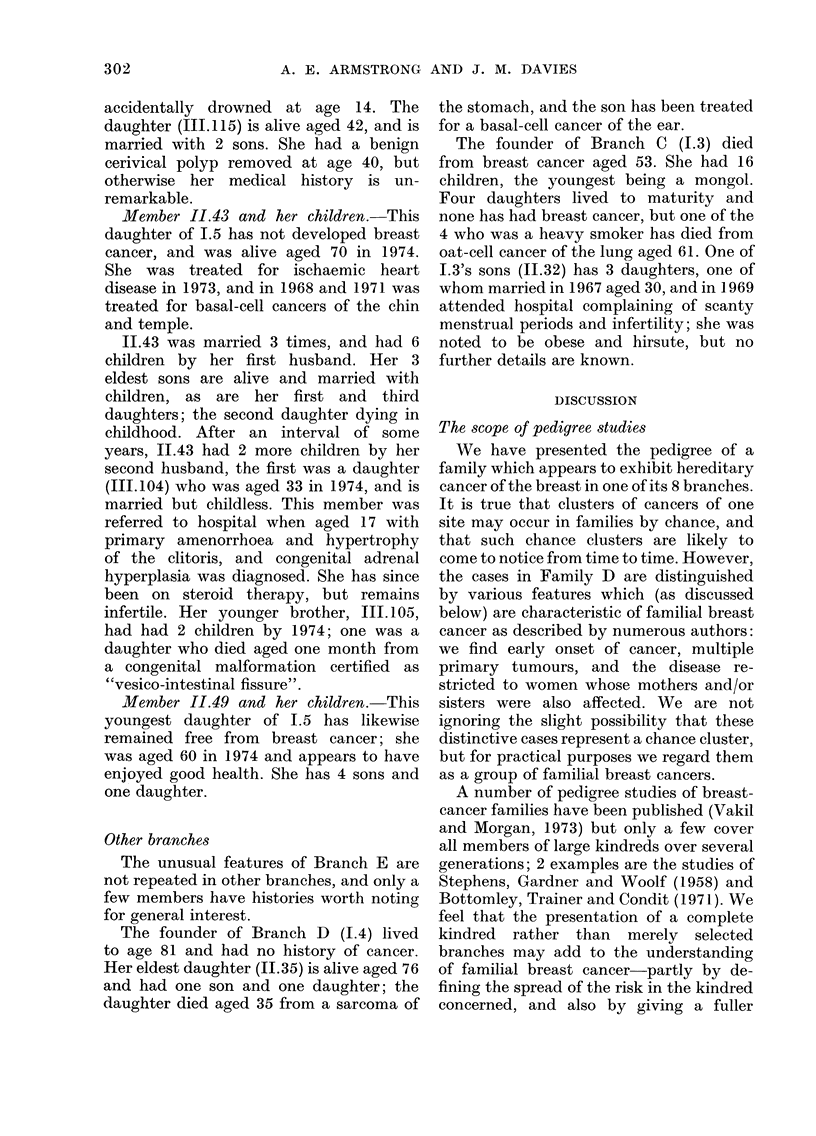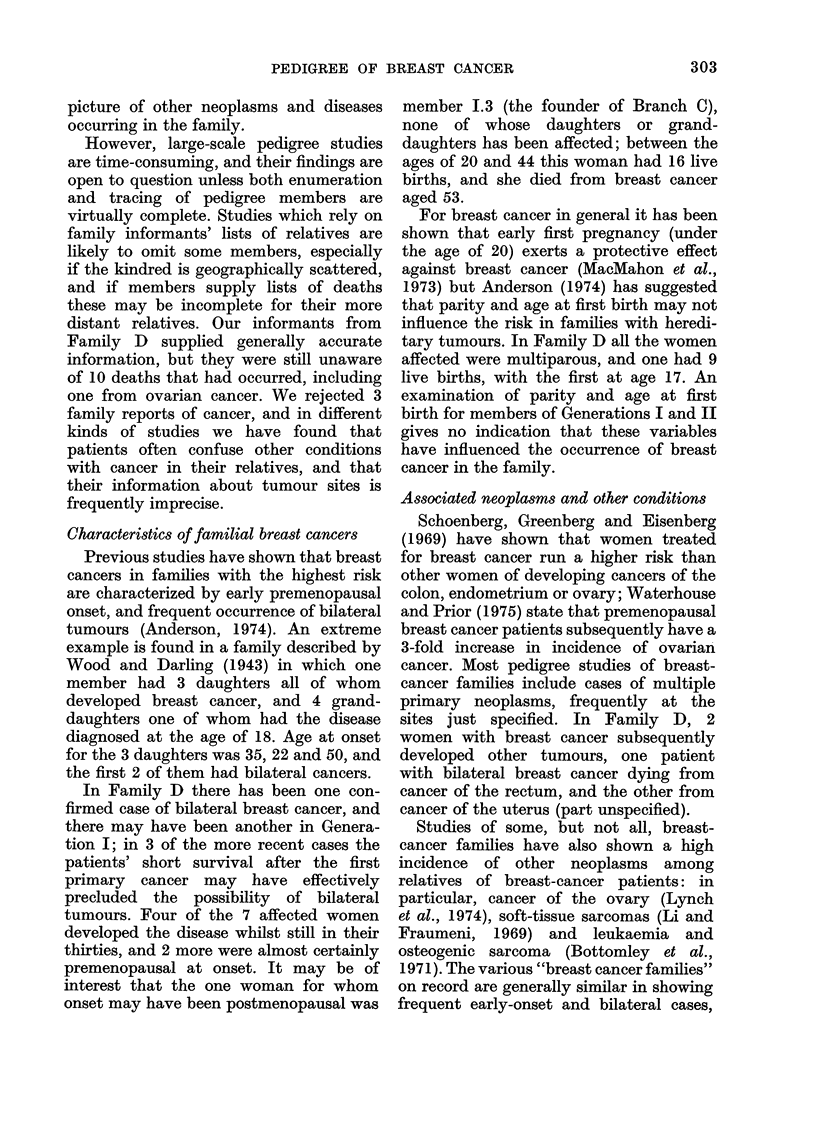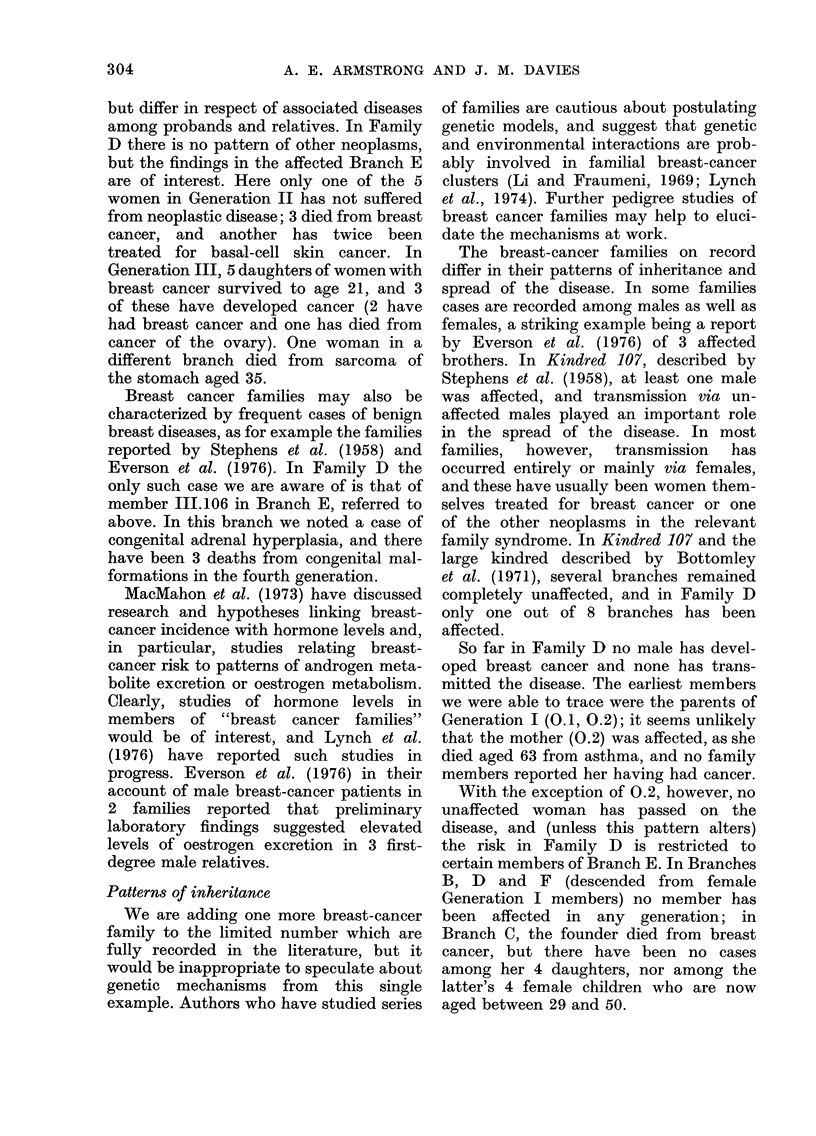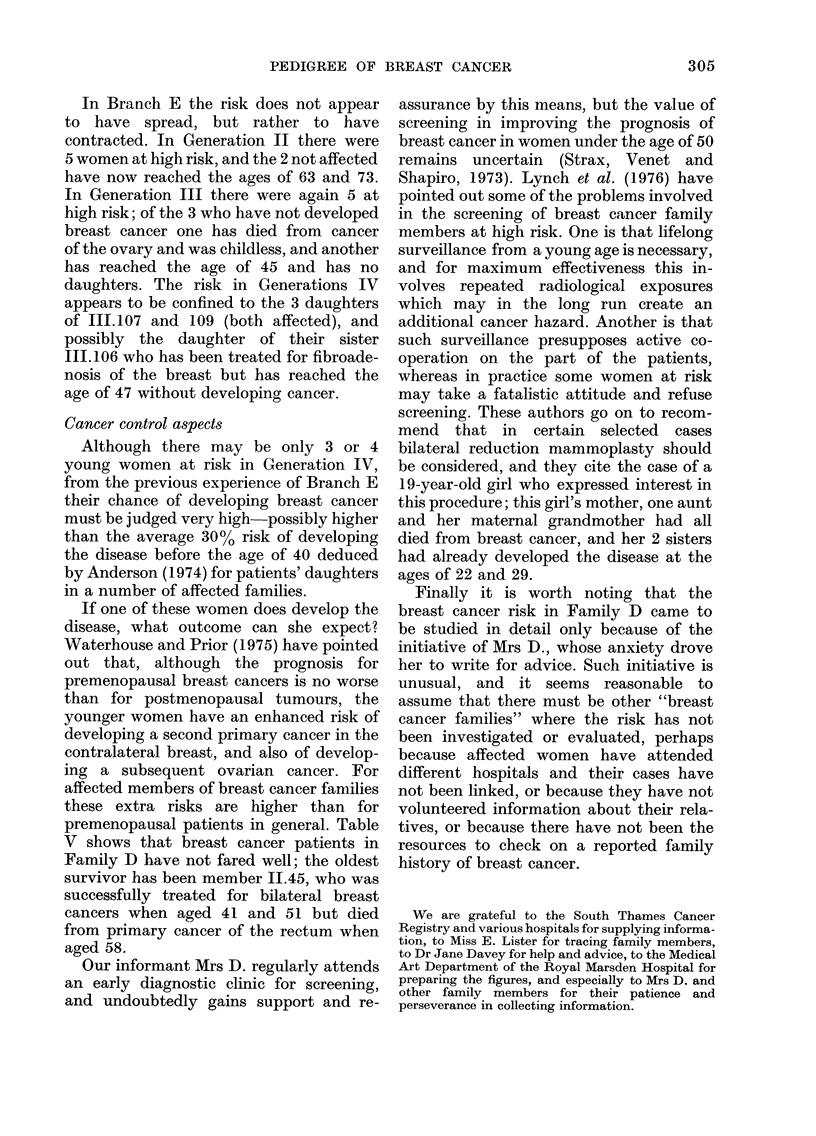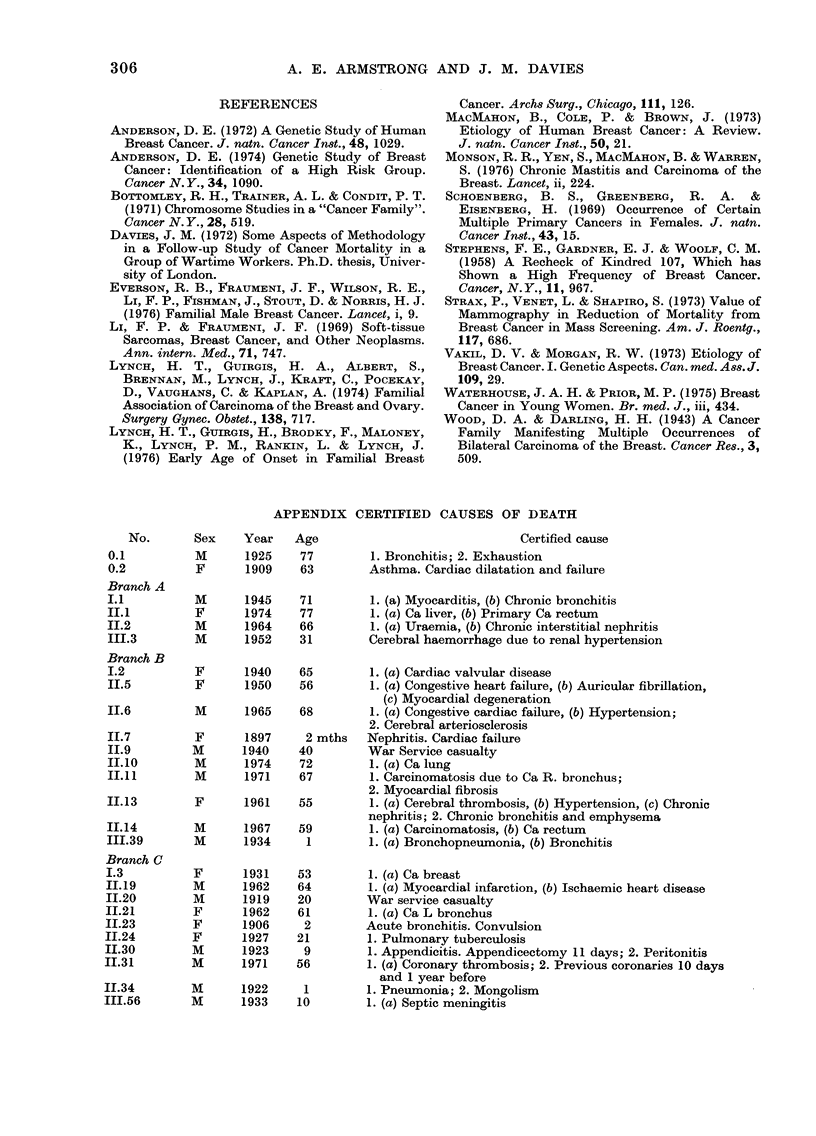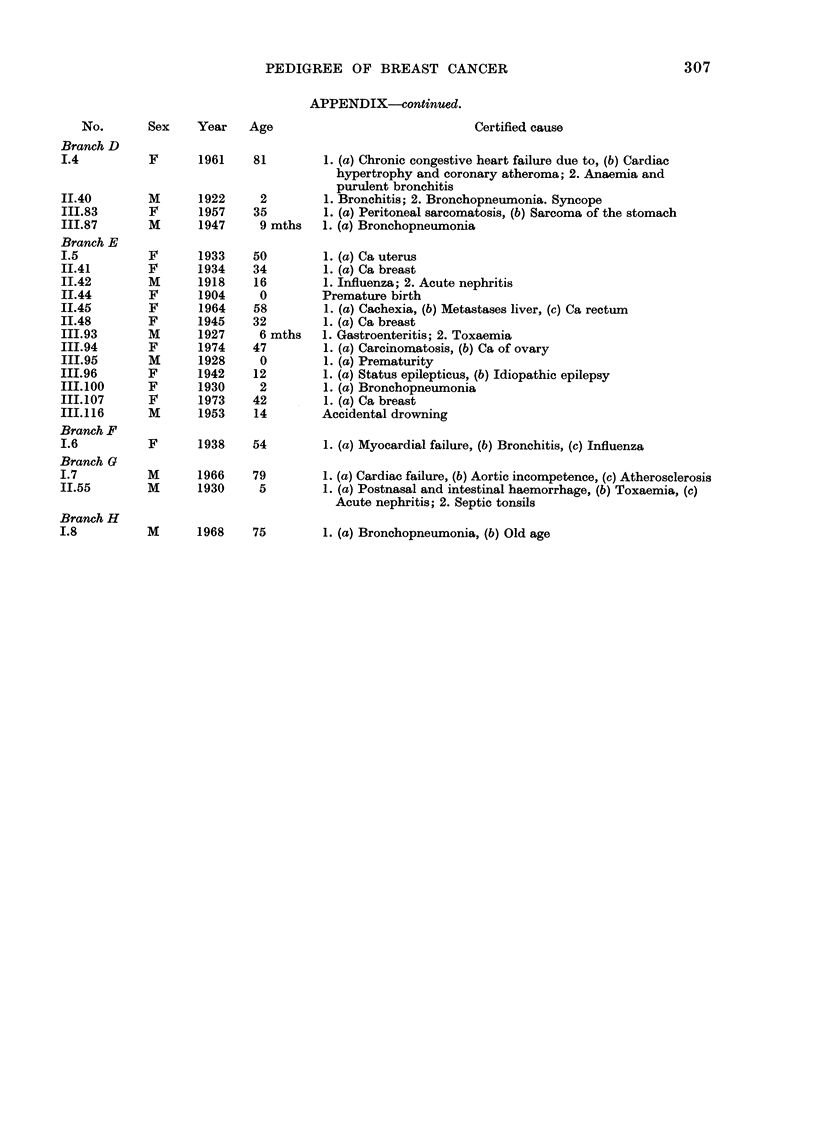# Familial breast cancer: report of a family pedigree.

**DOI:** 10.1038/bjc.1978.39

**Published:** 1978-02

**Authors:** A. E. Armstrong, J. M. Davies

## Abstract

Following a report of several relatives suffering from breast cancer, the occurrence of neoplasms in 3 generations of a large family was carefully checked. Members of one out of 8 branches were found to have a high incidence of breast cancer with 6 women affected, 4 of them under the age of 40. As well as early onset, these women presented other features typical of "breast cancer families": bilateral breast cancer, other second primary tumours, ovarian cancer in the daughter of one affected patient, and benign breast disease in the sister of another.


*Br. J. Cancer* (1978) **37**, 294

### FAMILIAL BREAST CANCER: REPORT OF A FAMILY PEDIGREE

A. E. ARMSTRONG* AND J. M. DAVIES

*From the Division of Epidemiology, Institute of Cancer Research, Clifton Avenue, Belmont, Sutton, Surrey*

Received 25 August 1977 Accepted 10 October 1977

**Summary.—Following a report of several relatives suffering from breast cancer, the occurrence of neoplasms in 3 generations of a large family was carefully checked. Members of one out of 8 branches were found to have a high incidence of breast cancer, with 6 women affected, 4 of them under the age of 40. As well as early onset, these women presented other features typical of "breast cancer families": bilateral breast cancer, other second primary tumours, ovarian cancer in the daughter of one affected patient, and benign breast disease in the sister of another.**

IN 1972, a woman in her early forties (Mrs D.) wrote to the Institute of Cancer Research expressing concern over the occurrence of 6 cases of breast cancer in her family; 3 of these cases were readily confirmed, and it was decided to study the pedigree of "Family D". An attempt was made to identify and trace all members of our informant's family starting with her maternal grandmother's parents, in order to ascertain all cases of cancer.

The literature describing a familial association for breast cancer is extensive, with reports of individual pedigrees and numerous case-control studies; most investigators have found a 2–3-fold increase in risk among relatives of breast cancer probands compared to women in matched control samples or in the general population (MacMahon, Cole and Brown, 1973; Vakil and Morgan, 1973). Recent work has shown that this increase in risk among relatives varies considerably when breast cancer probands are grouped by certain variables: there is a larger increase for relatives of probands characterized by premenopausal onset and/or bilateral disease, and in particular for sisters of probands whose mothers were also affected (Anderson, 1972, 1974).

Other variables shown to be correlated with breast cancer risk include age at first birth, age at menarché, age at natural or surgical menopause, and previous history of benign breast disease (MacMahon, Cole and Brown, 1973; Monson *et al.*, 1976).

### MATERIALS AND METHODS

### *Family D*

The study covers 3 generations of Family D, with the first comprising the informant's maternal grandmother and her siblings: 3 males and 5 females born between 1874 and 1894. Generation II includes the informant's mother, aunts and uncles, and their first cousins: these total 31 males and 26 females born during the period 1896–1925. Generation III comprises Mrs D herself, her siblings and her first and second cousins, 69 males and 64 females born between 1912 and 1958. There are numerous children of Generation III, but this fourth generation is still young and incomplete and has not been included. The family subdivides into 8 branches descended from respective members of Generation I; these branches are lettered

* Present address: Department of Community Medicine, University College Hospital Medical School, and Department of Cytology, University College Hospital, London WC1.

### PEDIGREE CHART

Fig. 1.

A–H in Fig. 1, which shows all the known pedigree members.

Most members of Family D have resided in and around 2 south coast towns ~10 miles apart. Their social background is indicated by the occupations of the male members as stated on various official records: all of Generation I and the majority of Generation II and III have been employed in unskilled occupations, although the later generations also include some semi-skilled and skilled manual workers.

The study was complicated by high rates of remarriage, common-law marriage and illegitimacy in some branches. This resulted in discrepancies in the surnames used in official records, making identification and tracing difficult and sometimes impossible. Another result was uncertainty about the paternity of some members of the family, and we therefore considered it inappropriate to collect mortality and morbidity data on the spouses of pedigree members.

Our aim was to identify all members of Family D, to trace their current mortality status, and to ascertain all cases of cancer.

*Identification of the pedigree members*

Our original informant, together with Generation II and III relatives from various branches, provided information on family membership, and this information was checked as thoroughly as possible. Wherever possible, we first followed the life history of each member of Generation I through the birth, marriage and death records of the Office of Population Censuses and Surveys (OPCS), and then checked on all children born to these members by searching the birth registers for a period from 2 years before each of their marriages up to a maternal age of 50 years. We followed the life histories of each member of Generation II in the same way, so identifying members of Generation III. However, this procedure could not be followed in a few cases, usually because members had very common surnames.

These checks were necessary in the first instance to obtain correct identifying details for family members, so that their cancer morbidity and mortality could be traced with confidence, and in the second instance to cover any members who had been omitted by our informants from their list of relatives. At the same time, the checks supplied accurate data on the number of live births and age at first live birth for each female pedigree member. We are reasonably certain that through the use of these methods our coverage of the pedigree membership is nearly complete; however, Generation III may be incomplete for Branches A and B because one Generation II member of each was lost to tracing, and these 2 members [II.3 (male); II.15 (female)] may have had unidentified children. We also cannot exclude the possibility that we have missed a few illegitimate children of male pedigree members.

*Tracing the pedigree members*

Reports were obtained from the family on whether relatives were alive or dead, and where they were living. These reports were verified where possible by reference to public records (electoral registers, town directories, birth, death and marriage registers, and miscellaneous sources), using the methods and criteria described by Davies (1972).

Table I shows the results of tracing up to the end of 1974, with each family member placed in one of the following 4 categories:

*Dead.*—Death certificate copies were obtained for all the 47 members traced as dead except for 2 men who died during war service. In no instance was there any doubt that a certificate related to the member being traced.

*Alive 1974, confirmed by records.*—All 119 members in this category were reported by the family to be alive at the end of 1974, and most were traced in public records up to 1973 or 1974.

*Alive 1974, unconfirmed by records.*—The 25 members in this category were reported by the family to be alive at the end of 1974, but for various reasons we were unable to trace them in public records for the previous 10 years. Death register searches were made for the period when there was no positive evidence that these members were still alive, but no relevant death references were found.

*Untraced.*—These 9 members have not been traced as alive after 1.1.65, and family contact with them has been lost. However, they have been sought without success in death registers and are likely to be still alive. Only 2 of the 57 members of Generation II have been lost sight of, both of these having moved away in the 1930s. In Genera-

TABLE I.—*Results of Tracing*

| Generation | Total members Male | Total members Female | Dead M | Dead F | Alive 1974 Confirmed M | Alive 1974 Confirmed F | Alive 1974 Unconfirmed M | Alive 1974 Unconfirmed F | Untraced M | Untraced F |
|---|---|---|---|---|---|---|---|---|---|---|
| 0 | 1 | 1 | 1 | 1 | — | — | — | — | — | — |
| I | 3 | 5 | 3 | 5 | — | — | — | — | — | — |
| II | 31 | 26 | 14 | 11 | 15 | 13 | 1 | 1 | 1 | 1 |
| III | 69 | 64 | 7 | 5 | 50 | 41 | 10 | 13 | 2 | 5 |
| Total | 104 | 96 | 25 | 22 | 65 | 54 | 11 | 14 | 3 | 6 |

tion III 7/133 are untraced: 5 women and 2 men from Branches A, C, D and E.

In assigning individuals to these categories we made considerable use of family reports which we found to be generally reliable. We were able to find records of all marriages and deaths reported by the family, and of all births except one (III.64): that of the daughter of a member who had moved away and whose married name presented difficulties. Only 10 deaths were omitted from the family reports: 6 childhood deaths, and 4 adult deaths amongst members with whom contact had been lost. In no case did we find the death of an individual whom the family had reported to be alive. We therefore felt able to place reliance on their reports that individual members were alive at specified dates, even in the absence of positive up-to-date confirmatory evidence from public records.

*Ascertainment of cancers*

Three main sources of information were available: death certificates, family reports, and Cancer Registry records from 1958 onwards. We were also able to obtain details from hospitals for some individuals, but in other instances old hospital case notes had been destroyed.

*Death certificates.*—These were available throughout the period covered by the study, and death was assigned to cancer on 13 certificates. However, the absence of any mention of cancer on a certificate does not exclude the occurrence of the disease, and 2 family members who had been treated for breast cancer had no mention of these tumours on their certificates.

*Cancer Registry records.*—The area where most family members have lived has been covered by the South Thames Cancer Registry (STCR) since 1958, though 1958–60 were developmental years. A list of family members was given to the Registry, which agreed to search for them in their alphabetical index of registrations. Cancer registrations were found for 9 members; 8 of these cases were already known to us from death certificates and family reports, but additional details such as dates of onset were obtained. The hitherto unknown case related to Member III.94 (see below) who was still alive at the time of ascertainment, but died during 1974. Only 2 known cases since 1958 were not found in the Registry records; one case of basal-cell cancer of the face, and one of lung cancer which had not been registered because the patient's hospital case notes had been lost.

*Family reports.*—Various members of the family reported histories of cancer in other members, usually in first-degree relatives such as mothers or sisters. The family reported 13 of the 16 individuals with confirmed cancer, and knew of both cancers diagnosed in Member II.45. They missed only 3 cases: 2 of skin cancer in members still living (II.43, III.82) and one of cancer of the ovary in Member III.94. This woman's mother (II.41) moved away on marriage and later died aged 34; our informants then lost touch with III.94 and her siblings, who were brought up by their father's family.

The family also reported 4 cancer histories for which we could not obtain firm medical confirmation. We were not able to accept 3 of these reports:

*I.4, Branch D.*—This woman died in 1961 aged 81, and we had a second-hand report that she had cancer of the rectum. However, a coroner's postmortem examination had been made, and the pathologist's report showed the intestinal tract to be normal and gave no indication of malignant disease at any site. Death was attributed to chronic heart disease.

*II.2, Branch A.*—We found no confirmation of a report from a cousin giving a history of cancer for this man without specifying the site. The death certificate issued in 1967 assigned death at age 66 to chronic interstitial nephritis, and there was no record of this individual in the Cancer Registry.

*I.6, Branch F.*—We were unable to confirm 2 separate but vague reports from nieces in different branches which suggested that this woman had had both breasts removed for cancer. We had difficulty in tracing this member, who had left the area and changed her surname, but we eventually found that she had died in 1938 aged 54, with death assigned to influenza, and without any mention of cancer on her death certificate. We made contact with this member's only daughter, but unfortunately she was extremely reticent, while denying any history of breast disease in her branch of the family. On balance we consider it unlikely that this member had breast cancer.

The case of cancer reported by the family which we have accepted in the absence of full medical confirmation relates to a female in Generation I (I.5, Branch E). This woman died from cancer of the uterus in 1933 aged 50; her one surviving daughter reported that she had earlier had both breasts removed. Hospital case notes dated 1957 for another daughter (now dead) also refer to a maternal history of mastectomy, stating "Mother died aged 53—?Ca. breast (1933). Had had mastectomy". We feel able to accept this as a case of breast cancer, especially since both informants were daughters and one of these has a consistent record of accurate reporting.

*Members with or without cancer*

A total of 16 individuals were found to have had cancer, but it was very difficult to establish with certainty that the other pedigree members were free from any history of the disease, the main limitation being the unavailability of comprehensive records prior to 1958. In this early period we had to rely on death certificates and family reports; cancers which were not assigned or contributory causes of death would not have appeared on death certificates, and some such cases might not have been known to our informants. However, we feel that breast cancer, with its characteristic treatment by mastectomy, would be less likely than cancer of some other sites to pass unnoticed by relatives, and of the 13 adult members who died before 1958 only one had lost contact with the family.

### RESULTS

Tables II and III show the numbers of male and female deaths in the 3 generations, and list those assigned to cancer; certified causes of all deaths are given in full in the Appendix.

The 3 males in Generation I died aged 71, 74 and 79; none is thought to have had cancer. Two females in this generation died from cancer; the other 3 died at ages 54, 65 and 81, and no evidence was found to support family reports that 2 of them had been treated for cancer.

TABLE II.—*Male Deaths*

| Generation and no. of members | | Deaths under 21 | Deaths over 21 | Deaths assigned to malignant neoplasms: Number, branch | Age at death | Site | Histology |
|---|---|---|---|---|---|---|---|
| I | 3 | 0 | 3 | — | — | — | |
| II | 31 | 6* | 8* | 10.B | 72 | Lung | Poorly differentiated carcinoma |
| | | | | 11.B | 67 | Bronchus | Not known |
| | | | | 14.B | 59 | Rectum | Squamous-cell carcinoma |
| III | 69 | 6 | 1 | — | — | — | |

* Including one death during war service.

TABLE III.—*Female Deaths*

| Generation and no. of members | | Deaths under 21 | Deaths over 21 | Deaths assigned to malignant neoplasms: Number, branch | Age at death | Site | Histology |
|---|---|---|---|---|---|---|---|
| I | 5 | 0 | 5 | 3.C | 53 | Breast | Not known |
| | | | | 5.E | 50 | Uterus* | Not known |
| II | 26 | 3 | 8 | 1.A | 77 | Rectum | Not known |
| | | | | 21.C | 61 | Bronchus | Oat-cell carcinoma |
| | | | | 41.E | 34 | Breast | Not known |
| | | | | 45.E | 58 | Rectum* | Adenocarcinoma |
| | | | | 48.E | 32 | Breast | Not known |
| III | 64 | 2 | 3 | 83.D | 35 | Stomach | Sarcoma |
| | | | | 94.E | 47 | Ovary | Well-differentiated solid adeno-carcinoma |
| | | | | 107.E | 42 | Breast | Trabecular-cell carcinoma |

* Previous history of breast cancer.

TABLE IV.—*Expected and Observed Deaths among Generation II Members from Age* 21 *to* 1974

| | No. | Person-years | Deaths from neoplasms: Exp. | Deaths from neoplasms: Obs. | Deaths from all other causes: Exp. | Deaths from all other causes: Obs. |
|---|---|---|---|---|---|---|
| Males | 25 | 1052 | 2·5 | 3 | 8·4 | 5 |
| Females | 23 | 896 | 1·4 | 5 | 4·0 | 3 |

One untraced male and one untraced female counted as alive.

For Generation II, expected deaths have been calculated by taking appropriate sex- and age-specific national death-rates for calendar periods from 1916, and applying these to the person-years of life experienced by members from age 21 to the end of 1974; the results are shown in Table IV. Male members have so far had a favourable mortality experience, and cancer deaths are not in excess; no non-fatal tumours have been ascertained. The family was selected for study on account of the unusual incidence of breast cancer, so that the excess of cancer deaths among females was predictable, but Generation II females have no excess mortality from other causes. One woman still living (II.43) has been treated for basal-cell cancer of the face.

Members of Generation III are still relatively young, with ages ranging from 16 to 62 in 1974. Only one adult male death and 3 adult female deaths have been ascertained, but the 3 female deaths were all from cancer. One male (III.82) has been treated for basal-cell cancer of the ear.

*Cases of breast cancer*

Table V gives details of all cases of breast cancer. Six of the 7 women with breast cancer are from Branch E of the pedigree, and Fig. 2 shows the relationship of these members; 2 of the 6 died from other cancers.

Details of age at onset and histology are available for only 3 individuals (all in Branch E):

*Member 11.45* was aged 41 when cancer of the right breast was diagnosed; the tumour was removed by radical mastectomy. When she was aged 51, cancer developed in the left breast, and she was treated by radical mastectomy

TABLE V.—*Cases of Breast Cancer*

| Pedigree number and branch | No. of live births | Age at 1st live birth | Age at onset of breast cancer | Age at death | Certified cause |
|---|---|---|---|---|---|
| I.3.C | 16 | 20 | Not known | 53 | Ca breast |
| I.5.E | 9 | 17 | Not known | 50 | Ca uterus |
| II.41.E | 5 | 26 | Not known | 34 | Ca breast |
| II.45.E | 4 | 23 | 41.Right breast<br>51.Left breast | 58 | Ca rectum |
| II.48.E | 2 | 20 | Not known | 32 | Ca breast |
| III.107.E | 3 | 28 | 38.Left breast | 42 | Ca breast |
| III.109.E | 3 | 21 | 35.Left breast | Alive | |

and postoperative radiotherapy. The second tumour had all the clinical features of a primary growth; there was an interval of nearly 10 years between the 2 cancers, the second tumour appeared in the upper outer quadrant of the left breast, and there were no signs of recurrence in the right breast. In both mastectomy specimens the tumour was of polygonal-cell type, and the axillary nodes were not involved. The patient died 7 years later aged 58 years from liver metastases from a primary carcinoma of the rectum.

*Member III.107* died from widespread metastases 4 years after the onset of a primary cancer in the left breast at the age of 38: a trabecular

FIG. 2.

carcinoma of high-grade malignancy. She had been treated by local mastectomy and postoperative radiotherapy.

*Member III.109* developed a primary carcinoma of the left breast at the age of 35. This was a localized mucoid carcinoma of moderate to high-grade malignancy, for which she was treated by local mastectomy and postoperative radiotherapy. She remains alive and well 2 years later.

Age at onset of breast cancer is not known for the other 4 women, but 2 died from the disease at the unusually early ages of 32 and 34. The other 2, aged 50 and 53 at death, were both reported by relatives to have undergone bilateral mastectomies. We could not obtain medical confirmation that bilateral tumours had occurred, but these reports (coupled with the fact that Member I.5 was certified as dying from another cancer) suggest the possibility that the first onset of breast cancer could have antedated death by some years in both cases.

*Branch E*

The main interest of the results is with Branch E, whose members are shown on Fig. 2. Most of these members have lived in the same town and have kept in touch with each other, the exception being the family of II.41, already referred to. The branch is particularly well-covered by STCR and hospital records.

*Member I.5 and her children.*—The founder of the branch, I.5, died aged 50 from cancer of the uterus (unspecified) but had previously been treated for breast cancer. She had 9 children: 3 sons and 6 daughters, including one pair of twins.

One son died from infectious disease aged 16, but the other 2 (II.46, II.47) were alive aged 64 and 66 in 1974; their medical histories appear unremarkable. II.46 has one daughter, and II.47 has one son and 3 daughters; there appears to be nothing of interest in the histories of these 5 Generation III members except that 2 of II.47's daughters have lost a child dying from congenital malformation.

For I.5's daughters the picture is different: one twin died at birth, and 3 had breast cancer; the other 2 are still alive. These daughters and their children will be considered in turn.

*Member II.41 and her children.*—This eldest daughter of I.5 died from breast cancer at the early age of 34, and was also reported by her daughter to have been epileptic. She had 3 sons and 2 daughters. Two sons died in infancy; the third son (III.97) is untraced, but was reported alive and well in 1965.

One of II.41's daughters died aged 12 from idiopathic epilepsy. The other, III.94, also suffered from grand mal epilepsy, and did not marry or have children. At the age of 36 she had a fimbrial cyst removed from the right fallopian tube. A year later cancer of the left ovary was diagnosed, and she was treated by total hysterectomy and bilateral salpingo-öophorectomy followed by radiotherapy. She died aged 47 from widespread metastases.

*Member II.45 and her children.*—This member had confirmed bilateral primary breast cancers, with first onset at age 41, and subsequently died aged 58 from primary cancer of the rectum. She had 3 daughters and one son who is alive and well.

II.45's eldest daughter is our informant, Mrs D., who is married with 3 children. In 1974, at the age of 44, she was treated for benign fibroadenosis of the breast; otherwise she has enjoyed good health, although she understandably suffers to some extent from cancerphobia.

II.45's second daughter developed breast cancer at the age of 38 and died 4 years later; she was married with 3 children. The youngest daughter was treated for breast cancer in 1973 when aged 35, but is alive and recurrence-free; she too has 3 children.

*Member II.48 and her children.*—This woman died from breast cancer at the early age of 32. She was married and had one son and one daughter; the son was accidentally drowned at age 14. The daughter (III.115) is alive aged 42, and is married with 2 sons. She had a benign cerivical polyp removed at age 40, but otherwise her medical history is unremarkable.

*Member II.43 and her children.*—This daughter of I.5 has not developed breast cancer, and was alive aged 70 in 1974. She was treated for ischaemic heart disease in 1973, and in 1968 and 1971 was treated for basal-cell cancers of the chin and temple.

II.43 was married 3 times, and had 6 children by her first husband. Her 3 eldest sons are alive and married with children, as are her first and third daughters; the second daughter dying in childhood. After an interval of some years, II.43 had 2 more children by her second husband, the first was a daughter (III.104) who was aged 33 in 1974, and is married but childless. This member was referred to hospital when aged 17 with primary amenorrhoea and hypertrophy of the clitoris, and congenital adrenal hyperplasia was diagnosed. She has since been on steroid therapy, but remains infertile. Her younger brother, III.105, had had 2 children by 1974; one was a daughter who died aged one month from a congenital malformation certified as "vesico-intestinal fissure".

*Member II.49 and her children.*—This youngest daughter of I.5 has likewise remained free from breast cancer; she was aged 60 in 1974 and appears to have enjoyed good health. She has 4 sons and one daughter.

### *Other branches*

The unusual features of Branch E are not repeated in other branches, and only a few members have histories worth noting for general interest.

The founder of Branch D (I.4) lived to age 81 and had no history of cancer. Her eldest daughter (II.35) is alive aged 76 and had one son and one daughter; the daughter died aged 35 from a sarcoma of the stomach, and the son has been treated for a basal-cell cancer of the ear.

The founder of Branch C (I.3) died from breast cancer aged 53. She had 16 children, the youngest being a mongol. Four daughters lived to maturity and none has had breast cancer, but one of the 4 who was a heavy smoker has died from oat-cell cancer of the lung aged 61. One of I.3's sons (II.32) has 3 daughters, one of whom married in 1967 aged 30, and in 1969 attended hospital complaining of scanty menstrual periods and infertility; she was noted to be obese and hirsute, but no further details are known.

### DISCUSSION

### *The scope of pedigree studies*

We have presented the pedigree of a family which appears to exhibit hereditary cancer of the breast in one of its 8 branches. It is true that clusters of cancers of one site may occur in families by chance, and that such chance clusters are likely to come to notice from time to time. However, the cases in Family D are distinguished by various features which (as discussed below) are characteristic of familial breast cancer as described by numerous authors: we find early onset of cancer, multiple primary tumours, and the disease restricted to women whose mothers and/or sisters were also affected. We are not ignoring the slight possibility that these distinctive cases represent a chance cluster, but for practical purposes we regard them as a group of familial breast cancers.

A number of pedigree studies of breast-cancer families have been published (Vakil and Morgan, 1973) but only a few cover all members of large kindreds over several generations; 2 examples are the studies of Stephens, Gardner and Woolf (1958) and Bottomley, Trainer and Condit (1971). We feel that the presentation of a complete kindred rather than merely selected branches may add to the understanding of familial breast cancer—partly by defining the spread of the risk in the kindred concerned, and also by giving a fuller picture of other neoplasms and diseases occurring in the family.

However, large-scale pedigree studies are time-consuming, and their findings are open to question unless both enumeration and tracing of pedigree members are virtually complete. Studies which rely on family informants' lists of relatives are likely to omit some members, especially if the kindred is geographically scattered, and if members supply lists of deaths these may be incomplete for their more distant relatives. Our informants from Family D supplied generally accurate information, but they were still unaware of 10 deaths that had occurred, including one from ovarian cancer. We rejected 3 family reports of cancer, and in different kinds of studies we have found that patients often confuse other conditions with cancer in their relatives, and that their information about tumour sites is frequently imprecise.

*Characteristics of familial breast cancers*

Previous studies have shown that breast cancers in families with the highest risk are characterized by early premenopausal onset, and frequent occurrence of bilateral tumours (Anderson, 1974). An extreme example is found in a family described by Wood and Darling (1943) in which one member had 3 daughters all of whom developed breast cancer, and 4 granddaughters one of whom had the disease diagnosed at the age of 18. Age at onset for the 3 daughters was 35, 22 and 50, and the first 2 of them had bilateral cancers.

In Family D there has been one confirmed case of bilateral breast cancer, and there may have been another in Generation I; in 3 of the more recent cases the patients' short survival after the first primary cancer may have effectively precluded the possibility of bilateral tumours. Four of the 7 affected women developed the disease whilst still in their thirties, and 2 more were almost certainly premenopausal at onset. It may be of interest that the one woman for whom onset may have been postmenopausal was member I.3 (the founder of Branch C), none of whose daughters or granddaughters has been affected; between the ages of 20 and 44 this woman had 16 live births, and she died from breast cancer aged 53.

For breast cancer in general it has been shown that early first pregnancy (under the age of 20) exerts a protective effect against breast cancer (MacMahon *et al.*, 1973) but Anderson (1974) has suggested that parity and age at first birth may not influence the risk in families with hereditary tumours. In Family D all the women affected were multiparous, and one had 9 live births, with the first at age 17. An examination of parity and age at first birth for members of Generations I and II gives no indication that these variables have influenced the occurrence of breast cancer in the family.

*Associated neoplasms and other conditions*

Schoenberg, Greenberg and Eisenberg (1969) have shown that women treated for breast cancer run a higher risk than other women of developing cancers of the colon, endometrium or ovary; Waterhouse and Prior (1975) state that premenopausal breast cancer patients subsequently have a 3-fold increase in incidence of ovarian cancer. Most pedigree studies of breast-cancer families include cases of multiple primary neoplasms, frequently at the sites just specified. In Family D, 2 women with breast cancer subsequently developed other tumours, one patient with bilateral breast cancer dying from cancer of the rectum, and the other from cancer of the uterus (part unspecified).

Studies of some, but not all, breast-cancer families have also shown a high incidence of other neoplasms among relatives of breast-cancer patients: in particular, cancer of the ovary (Lynch *et al.*, 1974), soft-tissue sarcomas (Li and Fraumeni, 1969) and leukaemia and osteogenic sarcoma (Bottomley *et al.*, 1971). The various "breast cancer families" on record are generally similar in showing frequent early-onset and bilateral cases, but differ in respect of associated diseases among probands and relatives. In Family D there is no pattern of other neoplasms, but the findings in the affected Branch E are of interest. Here only one of the 5 women in Generation II has not suffered from neoplastic disease; 3 died from breast cancer, and another has twice been treated for basal-cell skin cancer. In Generation III, 5 daughters of women with breast cancer survived to age 21, and 3 of these have developed cancer (2 have had breast cancer and one has died from cancer of the ovary). One woman in a different branch died from sarcoma of the stomach aged 35.

Breast cancer families may also be characterized by frequent cases of benign breast diseases, as for example the families reported by Stephens *et al.* (1958) and Everson *et al.* (1976). In Family D the only such case we are aware of is that of member III.106 in Branch E, referred to above. In this branch we noted a case of congenital adrenal hyperplasia, and there have been 3 deaths from congenital malformations in the fourth generation.

MacMahon *et al.* (1973) have discussed research and hypotheses linking breast-cancer incidence with hormone levels and, in particular, studies relating breast-cancer risk to patterns of androgen metabolite excretion or oestrogen metabolism. Clearly, studies of hormone levels in members of "breast cancer families" would be of interest, and Lynch *et al.* (1976) have reported such studies in progress. Everson *et al.* (1976) in their account of male breast-cancer patients in 2 families reported that preliminary laboratory findings suggested elevated levels of oestrogen excretion in 3 first-degree male relatives.

### *Patterns of inheritance*

We are adding one more breast-cancer family to the limited number which are fully recorded in the literature, but it would be inappropriate to speculate about genetic mechanisms from this single example. Authors who have studied series of families are cautious about postulating genetic models, and suggest that genetic and environmental interactions are probably involved in familial breast-cancer clusters (Li and Fraumeni, 1969; Lynch *et al.*, 1974). Further pedigree studies of breast cancer families may help to elucidate the mechanisms at work.

The breast-cancer families on record differ in their patterns of inheritance and spread of the disease. In some families cases are recorded among males as well as females, a striking example being a report by Everson *et al.* (1976) of 3 affected brothers. In *Kindred 107*, described by Stephens *et al.* (1958), at least one male was affected, and transmission *via* unaffected males played an important role in the spread of the disease. In most families, however, transmission has occurred entirely or mainly *via* females, and these have usually been women themselves treated for breast cancer or one of the other neoplasms in the relevant family syndrome. In *Kindred 107* and the large kindred described by Bottomley *et al.* (1971), several branches remained completely unaffected, and in Family D only one out of 8 branches has been affected.

So far in Family D no male has developed breast cancer and none has transmitted the disease. The earliest members we were able to trace were the parents of Generation I (O.1, O.2); it seems unlikely that the mother (O.2) was affected, as she died aged 63 from asthma, and no family members reported her having had cancer.

With the exception of O.2, however, no unaffected woman has passed on the disease, and (unless this pattern alters) the risk in Family D is restricted to certain members of Branch E. In Branches B, D and F (descended from female Generation I members) no member has been affected in any generation; in Branch C, the founder died from breast cancer, but there have been no cases among her 4 daughters, nor among the latter's 4 female children who are now aged between 29 and 50.

In Branch E the risk does not appear to have spread, but rather to have contracted. In Generation II there were 5 women at high risk, and the 2 not affected have now reached the ages of 63 and 73. In Generation III there were again 5 at high risk; of the 3 who have not developed breast cancer one has died from cancer of the ovary and was childless, and another has reached the age of 45 and has no daughters. The risk in Generations IV appears to be confined to the 3 daughters of III.107 and 109 (both affected), and possibly the daughter of their sister III.106 who has been treated for fibroadenosis of the breast but has reached the age of 47 without developing cancer.

*Cancer control aspects*

Although there may be only 3 or 4 young women at risk in Generation IV, from the previous experience of Branch E their chance of developing breast cancer must be judged very high—possibly higher than the average 30% risk of developing the disease before the age of 40 deduced by Anderson (1974) for patients' daughters in a number of affected families.

If one of these women does develop the disease, what outcome can she expect? Waterhouse and Prior (1975) have pointed out that, although the prognosis for premenopausal breast cancers is no worse than for postmenopausal tumours, the younger women have an enhanced risk of developing a second primary cancer in the contralateral breast, and also of developing a subsequent ovarian cancer. For affected members of breast cancer families these extra risks are higher than for premenopausal patients in general. Table V shows that breast cancer patients in Family D have not fared well; the oldest survivor has been member II.45, who was successfully treated for bilateral breast cancers when aged 41 and 51 but died from primary cancer of the rectum when aged 58.

Our informant Mrs D. regularly attends an early diagnostic clinic for screening, and undoubtedly gains support and reassurance by this means, but the value of screening in improving the prognosis of breast cancer in women under the age of 50 remains uncertain (Strax, Venet and Shapiro, 1973). Lynch *et al.* (1976) have pointed out some of the problems involved in the screening of breast cancer family members at high risk. One is that lifelong surveillance from a young age is necessary, and for maximum effectiveness this involves repeated radiological exposures which may in the long run create an additional cancer hazard. Another is that such surveillance presupposes active cooperation on the part of the patients, whereas in practice some women at risk may take a fatalistic attitude and refuse screening. These authors go on to recommend that in certain selected cases bilateral reduction mammoplasty should be considered, and they cite the case of a 19-year-old girl who expressed interest in this procedure; this girl's mother, one aunt and her maternal grandmother had all died from breast cancer, and her 2 sisters had already developed the disease at the ages of 22 and 29.

Finally it is worth noting that the breast cancer risk in Family D came to be studied in detail only because of the initiative of Mrs D., whose anxiety drove her to write for advice. Such initiative is unusual, and it seems reasonable to assume that there must be other "breast cancer families" where the risk has not been investigated or evaluated, perhaps because affected women have attended different hospitals and their cases have not been linked, or because they have not volunteered information about their relatives, or because there have not been the resources to check on a reported family history of breast cancer.

We are grateful to the South Thames Cancer Registry and various hospitals for supplying information, to Miss E. Lister for tracing family members, to Dr Jane Davey for help and advice, to the Medical Art Department of the Royal Marsden Hospital for preparing the figures, and especially to Mrs D. and other family members for their patience and perseverance in collecting information.

### REFERENCES

ANDERSON, D. E. (1972) A Genetic Study of Human Breast Cancer. *J. natn. Cancer Inst.*, **48**, 1029.

ANDERSON, D. E. (1974) Genetic Study of Breast Cancer: Identification of a High Risk Group. *Cancer N.Y.*, **34**, 1090.

BOTTOMLEY, R. H., TRAINER, A. L. & CONDIT, P. T. (1971) Chromosome Studies in a "Cancer Family". *Cancer N.Y.*, **28**, 519.

DAVIES, J. M. (1972) Some Aspects of Methodology in a Follow-up Study of Cancer Mortality in a Group of Wartime Workers. Ph.D. thesis, University of London.

EVERSON, R. B., FRAUMENI, J. F., WILSON, R. E., LI, F. P., FISHMAN, J., STOUT, D. & NORRIS, H. J. (1976) Familial Male Breast Cancer. *Lancet*, i, 9.

LI, F. P. & FRAUMENI, J. F. (1969) Soft-tissue Sarcomas, Breast Cancer, and Other Neoplasms. *Ann. intern. Med.*, **71**, 747.

LYNCH, H. T., GUIRGIS, H. A., ALBERT, S., BRENNAN, M., LYNCH, J., KRAFT, C., POCEKAY, D., VAUGHANS, C. & KAPLAN, A. (1974) Familial Association of Carcinoma of the Breast and Ovary. *Surgery Gynec. Obstet.*, **138**, 717.

LYNCH, H. T., GUIRGIS, H., BRODKY, F., MALONEY, K., LYNCH, P. M., RANKIN, L. & LYNCH, J. (1976) Early Age of Onset in Familial Breast Cancer. *Archs Surg., Chicago*, **111**, 126.

MACMAHON, B., COLE, P. & BROWN, J. (1973) Etiology of Human Breast Cancer: A Review. *J. natn. Cancer Inst.*, **50**, 21.

MONSON, R. R., YEN, S., MACMAHON, B. & WARREN, S. (1976) Chronic Mastitis and Carcinoma of the Breast. *Lancet*, ii, 224.

SCHOENBERG, B. S., GREENBERG, R. A. & EISENBERG, H. (1969) Occurrence of Certain Multiple Primary Cancers in Females. *J. natn. Cancer Inst.*, **43**, 15.

STEPHENS, F. E., GARDNER, E. J. & WOOLF, C. M. (1958) A Recheck of Kindred 107, Which has Shown a High Frequency of Breast Cancer. *Cancer, N.Y.*, **11**, 967.

STRAX, P., VENET, L. & SHAPIRO, S. (1973) Value of Mammography in Reduction of Mortality from Breast Cancer in Mass Screening. *Am. J. Roentg.*, **117**, 686.

VAKIL, D. V. & MORGAN, R. W. (1973) Etiology of Breast Cancer. I. Genetic Aspects. *Can. med. Ass. J.* **109**, 29.

WATERHOUSE, J. A. H. & PRIOR, M. P. (1975) Breast Cancer in Young Women. *Br. med. J.*, iii, 434.

WOOD, D. A. & DARLING, H. H. (1943) A Cancer Family Manifesting Multiple Occurrences of Bilateral Carcinoma of the Breast. *Cancer Res.*, **3**, 509.

### APPENDIX CERTIFIED CAUSES OF DEATH

| No. | Sex | Year | Age | Certified cause |
|---|---|---|---|---|
| 0.1 | M | 1925 | 77 | 1. Bronchitis; 2. Exhaustion |
| 0.2 | F | 1909 | 63 | Asthma. Cardiac dilatation and failure |
| *Branch A* | | | | |
| I.1 | M | 1945 | 71 | 1. (a) Myocarditis, (*b*) Chronic bronchitis |
| II.1 | F | 1974 | 77 | 1. (*a*) Ca liver, (*b*) Primary Ca rectum |
| II.2 | M | 1964 | 66 | 1. (*a*) Uraemia, (*b*) Chronic interstitial nephritis |
| III.3 | M | 1952 | 31 | Cerebral haemorrhage due to renal hypertension |
| *Branch B* | | | | |
| I.2 | F | 1940 | 65 | 1. (*a*) Cardiac valvular disease |
| II.5 | F | 1950 | 56 | 1. (*a*) Congestive heart failure, (*b*) Auricular fibrillation, (*c*) Myocardial degeneration |
| II.6 | M | 1965 | 68 | 1. (*a*) Congestive cardiac failure, (*b*) Hypertension; 2. Cerebral arteriosclerosis |
| II.7 | F | 1897 | 2 mths | Nephritis. Cardiac failure |
| II.9 | M | 1940 | 40 | War Service casualty |
| II.10 | M | 1974 | 72 | 1. (*a*) Ca lung |
| II.11 | M | 1971 | 67 | 1. Carcinomatosis due to Ca R. bronchus; 2. Myocardial fibrosis |
| II.13 | F | 1961 | 55 | 1. (*a*) Cerebral thrombosis, (*b*) Hypertension, (*c*) Chronic nephritis; 2. Chronic bronchitis and emphysema |
| II.14 | M | 1967 | 59 | 1. (*a*) Carcinomatosis, (*b*) Ca rectum |
| III.39 | M | 1934 | 1 | 1. (*a*) Bronchopneumonia, (*b*) Bronchitis |
| *Branch C* | | | | |
| I.3 | F | 1931 | 53 | 1. (*a*) Ca breast |
| II.19 | M | 1962 | 64 | 1. (*a*) Myocardial infarction, (*b*) Ischaemic heart disease |
| II.20 | M | 1919 | 20 | War service casualty |
| II.21 | F | 1962 | 61 | 1. (*a*) Ca L bronchus |
| II.23 | F | 1906 | 2 | Acute bronchitis. Convulsion |
| II.24 | F | 1927 | 21 | 1. Pulmonary tuberculosis |
| II.30 | M | 1923 | 9 | 1. Appendicitis. Appendicectomy 11 days; 2. Peritonitis |
| II.31 | M | 1971 | 56 | 1. (*a*) Coronary thrombosis; 2. Previous coronaries 10 days and 1 year before |
| II.34 | M | 1922 | 1 | 1. Pneumonia; 2. Mongolism |
| III.56 | M | 1933 | 10 | 1. (*a*) Septic meningitis |

APPENDIX—*continued.*

| No. | Sex | Year | Age | Certified cause |
|---|---|---|---|---|
| *Branch D* | | | | |
| I.4 | F | 1961 | 81 | 1. (*a*) Chronic congestive heart failure due to, (*b*) Cardiac hypertrophy and coronary atheroma; 2. Anaemia and purulent bronchitis |
| II.40 | M | 1922 | 2 | 1. Bronchitis; 2. Bronchopneumonia. Syncope |
| III.83 | F | 1957 | 35 | 1. (*a*) Peritoneal sarcomatosis, (*b*) Sarcoma of the stomach |
| III.87 | M | 1947 | 9 mths | 1. (*a*) Bronchopneumonia |
| *Branch E* | | | | |
| I.5 | F | 1933 | 50 | 1. (*a*) Ca uterus |
| II.41 | F | 1934 | 34 | 1. (*a*) Ca breast |
| II.42 | M | 1918 | 16 | 1. Influenza; 2. Acute nephritis |
| II.44 | F | 1904 | 0 | Premature birth |
| II.45 | F | 1964 | 58 | 1. (*a*) Cachexia, (*b*) Metastases liver, (*c*) Ca rectum |
| II.48 | F | 1945 | 32 | 1. (*a*) Ca breast |
| III.93 | M | 1927 | 6 mths | 1. Gastroenteritis; 2. Toxaemia |
| III.94 | F | 1974 | 47 | 1. (*a*) Carcinomatosis, (*b*) Ca of ovary |
| III.95 | M | 1928 | 0 | 1. (*a*) Prematurity |
| III.96 | F | 1942 | 12 | 1. (*a*) Status epilepticus, (*b*) Idiopathic epilepsy |
| III.100 | F | 1930 | 2 | 1. (*a*) Bronchopneumonia |
| III.107 | F | 1973 | 42 | 1. (*a*) Ca breast |
| III.116 | M | 1953 | 14 | Accidental drowning |
| *Branch F* | | | | |
| I.6 | F | 1938 | 54 | 1. (*a*) Myocardial failure, (*b*) Bronchitis, (*c*) Influenza |
| *Branch G* | | | | |
| I.7 | M | 1966 | 79 | 1. (*a*) Cardiac failure, (*b*) Aortic incompetence, (*c*) Atherosclerosis |
| II.55 | M | 1930 | 5 | 1. (*a*) Postnasal and intestinal haemorrhage, (*b*) Toxaemia, (*c*) Acute nephritis; 2. Septic tonsils |
| *Branch H* | | | | |
| I.8 | M | 1968 | 75 | 1. (*a*) Bronchopneumonia, (*b*) Old age |